# Attentive pairwise interaction network for AI-assisted clock drawing test assessment of early visuospatial deficits

**DOI:** 10.1038/s41598-023-44723-1

**Published:** 2023-10-23

**Authors:** Raksit Raksasat, Surat Teerapittayanon, Sirawaj Itthipuripat, Kearkiat Praditpornsilpa, Aisawan Petchlorlian, Thiparat Chotibut, Chaipat Chunharas, Itthi Chatnuntawech

**Affiliations:** 1 Computational Molecular Biology Group, Faculty of Medicine, Chulalongkorn University, Bangkok, Thailand; 2 National Nanotechnology Center, National Science and Technology Development Agency, Pathum Thani, Thailand; 3 Neuroscience Center for Research and Innovation, Learning Institute, King Mongkut’s University of Technology Thonburi, Bangkok, Thailand; 4 Big Data Experience Center, King Mongkut’s University of Technology Thonburi, Bangkok, Thailand; 5 Geriatric Excellence Center, King Chulalongkorn Memorial Hospital, Thai Red Cross Society, Faculty of Medicine, Chulalongkorn University, Bangkok, Thailand; 6 Chula Intelligent and Complex Systems Lab, Department of Physics, Faculty of Science, Chulalongkorn University, Bangkok, Thailand; 7 Chula Neuroscience Center, King Chulalongkorn Memorial Hospital, Thai Red Cross Society, Bangkok, Thailand; 8 Cognitive Clinical and Computational Neuroscience, Department of Internal Medicine, Faculty of Medicine, Chulalongkorn University, Bangkok, Thailand

**Keywords:** Computer science, Cognitive neuroscience, Neurology

## Abstract

Dementia is a debilitating neurological condition which impairs the cognitive function and the ability to take care of oneself. The Clock Drawing Test (CDT) is widely used to detect dementia, but differentiating normal from borderline cases requires years of clinical experience. Misclassifying mild abnormal as normal will delay the chance to investigate for potential reversible causes or slow down the progression. To help address this issue, we propose an automatic CDT scoring system that adopts Attentive Pairwise Interaction Network (API-Net), a fine-grained deep learning model that is designed to distinguish visually similar images. Inspired by how humans often learn to recognize different objects by looking at two images side-by-side, API-Net is optimized using image pairs in a contrastive manner, as opposed to standard supervised learning, which optimizes a model using individual images. In this study, we extend API-Net to infer Shulman CDT scores from a dataset of 3108 subjects. We compare the performance of API-Net to that of convolutional neural networks: VGG16, ResNet-152, and DenseNet-121. The best API-Net achieves an F1-score of 0.79, which is a 3% absolute improvement over ResNet-152’s F1-score of 0.76. The code for API-Net and the dataset used have been made available at https://github.com/cccnlab/CDT-API-Network.

## Introduction

Dementia affects millions of people worldwide^1^, and early detection is essential for deterring disease progression since there is currently no curative treatment available^2,3^. Standard neuropsychological screening tests such as the Mini-Mental State Examination (MMSE)^4^ and the Montreal Cognitive Assessment (MoCA)^5^ have been developed for early detection, but they are time-consuming and require trained personnel to administer and interpret, making them difficult to administer in remote areas.

The clock-drawing test (CDT) is a classic bedside test for cognitive screening and monitoring the severity of dementia with high test-retest and inter-rater reliability^6–8^. While CDT is relatively easy to administer, evaluating and interpreting the clock drawing is not straightforward and typically requires clinical expertise. Several scoring systems have been developed and adopted to aid clock drawing image evaluation^8–10^, including the well-accepted Shulman scoring system^7,11,12^. According to the Shulman scoring rubric^7^, a clock drawing image receives a score ranging from zero to five, with higher scores representing fewer drawing errors and, hence, potentially inferring better cognitive function. Nevertheless, the same clock drawing image may still receive different scores from different scorers, depending on how they interpret the rubric, especially between normal clock drawings and drawings with minor errors. Consequently, experienced scorers with clinical expertise are typically required, which is often impractical in many circumstances, necessitating the development of a more consistent and objective alternative.

To enable more consistent and less subjective CDT assessments, several studies have proposed machine learning pipelines that digitize clock drawing data, extract geometrical and/or temporal features from the data, and train models in a supervised manner^13–18^. While such pipelines have demonstrated promising performance on the tasks, they heavily rely on human-designed features, which have been shown to be suboptimal in a wide range of tasks such as the ImageNet large-scale visual recognition^19,20^. Consequently, deep learning, which does not require human-designed features and has significantly produced cutting-edge outcomes in image classification tasks^19,21–24^, has been adopted for improved CDT assessments^25–32^. Despite their ability to make accurate predictions, these deep-learning-based techniques still make errors in difficult cases. For instance, for the Shulman scoring task, the majority of the errors were due to the misclassification of adjacent clock scores where the clock images were visually similar^27^, limiting a direct application of such methods to the detection of early dementia and milder neurocognitive disorders such as mild cognitive impairment (MCI).

In this work, we reformulated the clock drawing image scoring task as fine-grained image classification and proposed a deep learning framework that aims to alleviate the adjacent clock score misclassification problem, especially between normal clock drawings and drawings with minor errors. In particular, we extended the training scheme proposed in the Attentive Pairwise Interaction Network (API-Net)^33^ to the Shulman clock scoring task^7^. Under the proposed training scheme, which draws inspiration from how humans frequently compare two images side-by-side to detect subtle differences^34^, we present image pairs to a deep learning model and encourage the model to come up with data representation that can later be used to provide clock scores, based on the information from both images in a pair in a contrastive manner. This is in contrast to all prior works which only train their models to make predictions without any comparative interactions^25–31^. To assess the performance gained from the proposed training scheme, we compared API-Net to widely-used models consisting of VGG16^21^, ResNet-152^22^, and DenseNet-121^23^, which have established state-of-the-art performance in the Shulman scoring task^27^. API-Net has shown an improvement in F1-score of 3% over traditional methods. Specifically, API-Net with ResNet-152 backbone achieves an F1-score of 0.79, which is higher than ResNet-152’s F1-score of 0.76. In the spirit of reproducibility, we have made our dataset and accompanying code available at https://github.com/cccnlab/CDT-API-Network.

## Methods

### Data acquisition and evaluation

**Figure 1:**
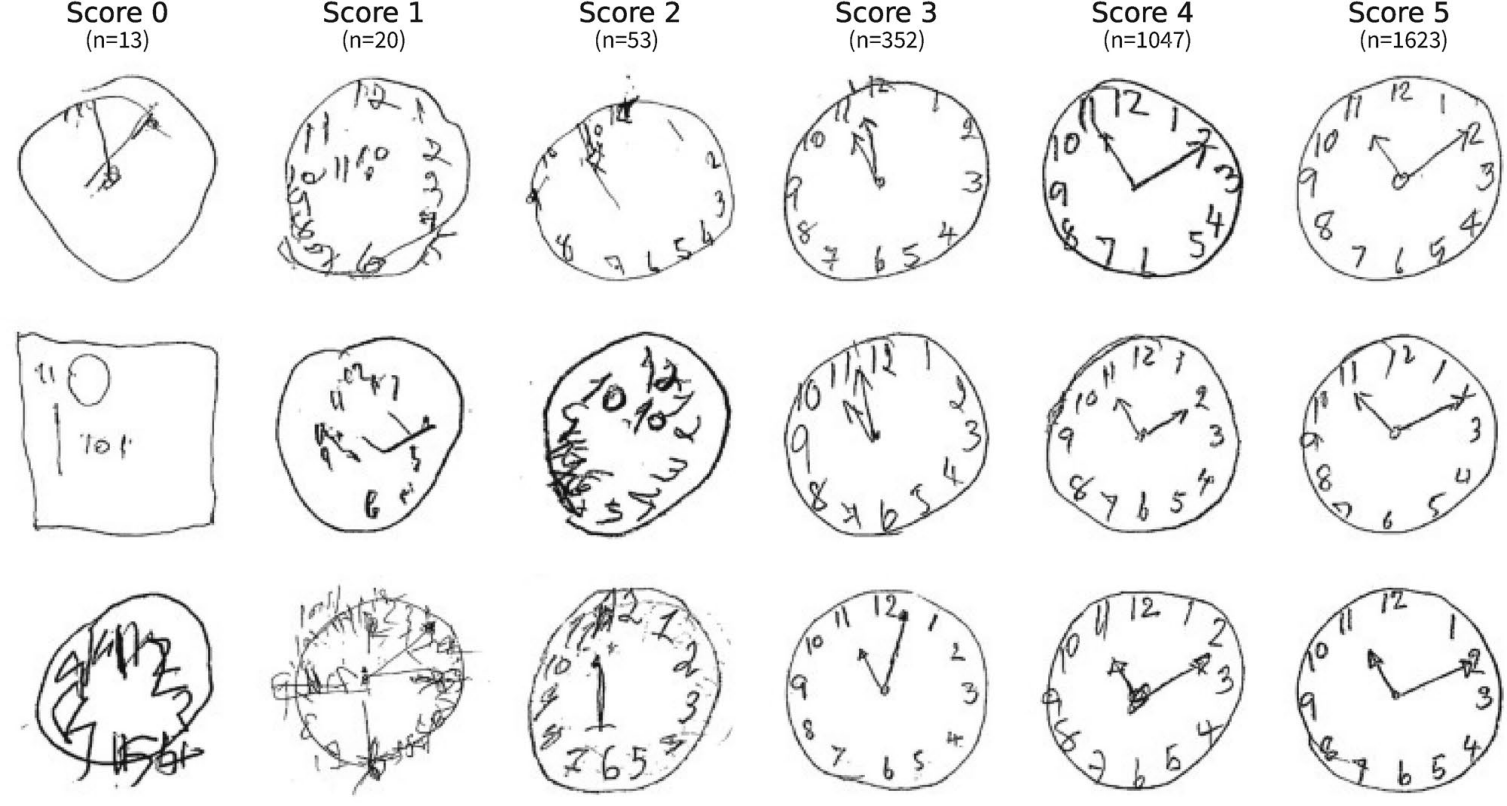
Example clock drawing images with the Shulman scores ranging from zero to five with lower scores indicating more severe cognitive impairment. The number of images in each score is shown below the score label.

Clock drawing images were collected as part of the paper-based MoCA assessments during 2019–2021 at the healthy aging cohort and neurology outpatient clinic of the King Chulalongkorn Memorial Hospital, Bangkok, Thailand, with institutional ethics committee approval (0926/64). The participants’ ages ranged from 29 to 90 with the median age of 67, and the female-to-male ratio was 3:1. For the clock drawing part of the MoCA assessment, we instructed the participants to draw a circular clock with all the numbers and the clock hands specifying the time of 11:10. The MoCA results were scanned, anonymized, and cropped to include only the clock drawing part using an in-house software. All the images with extra information not provided by the participants, such as clinical notes from the test administers, were excluded, resulting in a total of 3108 images. Then, the images were given scores according to the Shulman scoring rubric^7^ by a majority vote between three experienced neuropsychologists/neurologists, which were then used for model training. The Shulman score classifies each clock drawing into 6 groups depending on the types of abnormality and readability of the drawing (5 = normal, 4 = minor visuo-spatial deficits, 3 = incorrect representation of the correct time, 2 = moderate visuo-spatial deficits, 1 = severe visuo-spatial deficits, 0 = No reasonable depiction of a clock)^7^. The number of images for each score and example images are shown in Fig. 1.

### Image preprocessing and augmentation

The acquired clock drawing images were resized to 256 × 256 pixels. Image augmentation was then applied to increase the diversity of the data used for more effective model training. Specifically, we transformed the acquired images by varying image brightness, contrast, shift and scale. Unlike previous works^27,31^, we did not rotate or flip any clock images in our augmentation pipeline since these transformations could corrupt the semantics of a clock, which are essential for accurate clock scoring. After the augmentation process, the images were standardized using the mean and standard deviations from the ImageNet dataset^19^.

### Clock drawing API-Net architecture

**Figure 2:**
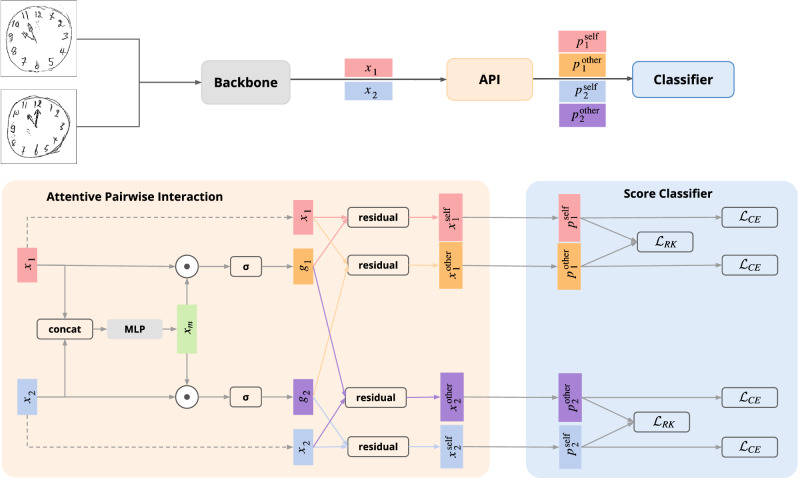
The API-Net architecture. The model consists of three components. The *backbone* component is used to extract the embedding vector of each input image. The *Attentive Pairwise Interaction (API)* component takes the embedding vectors of the image pair as its inputs, creates a mutual vector ${x}_{m}$, and uses ${x}_{m}$ to generate the gate vectors. Then, it computes four attentive feature vectors, ${x}^{\text {self}}_{1}$, ${x}^{\text {other}}_{1}$, ${x}^{\text {self}}_{2}$, and ${x}^{\text {other}}_{2}$. Finally, the *classifier* component uses the resulting vectors to predict the Shulman scores. These predicted scores are used to compute the loss function ${\mathcal {L}}$ for optimizing the model’s parameters.

We reformulated the clock drawing image scoring task as fine-grained image classification and solved it using API-Net^33^, in contrast to a previous work that relies on the conventional non-contrastive classification pipeline^27^. Inspired by how humans often compare image pairs to detect subtle differences in images that appear similar^34^, API-Net is trained using image pairs in a contrastive manner. API-Net consists of three components: a backbone, an API component, and a classifier. For the training phase, the backbone, the API component, and the classifier are connected sequentially as shown in Fig. 2, whereas the API component is not included in the test phase.

The backbone component is a convolutional neural network (CNN) with the final classification layer(s) removed. The backbone acts as a feature extractor that takes in a pair of visually similar clock drawing images as input and generates two embedding vectors, $x_1$ and $x_2$, one for each image in the pair. The API component then processes the two embedding vectors based on attentive pairwise interaction and output four feature vectors that are used by the classifier to produce the final clock scores for the pair. In particular, the two embedding vectors are concatenated and processed by a multilayer perceptron (MLP) that consists of two fully connected layers with a dropout layer^35^ in between, producing the mutual vector ${x}_{m}$ that potentially contains contrastive details of the image pairs. The gate vector is then computed as follows:

1 $$\begin{aligned} \begin{gathered} {g}_{i} = \text {sigmoid}({x}_{m}\odot {x}_{i}),\quad i\in \{1,2\},\\ \end{gathered} \end{aligned}$$

where $\odot$ is the Hadamard (element-wise) product. Using both the gate and original embedding vectors, four attentive feature vectors are calculated through the residual attention mechanism,

2 $$\begin{aligned} {x}^{\text {self}}_{1} = {x}_{1} + {x}_{1}\odot {g}_{1}, \nonumber \\ {x}^{\text {self}}_{2} = {x}_{2} + {x}_{2}\odot {g}_{2}, \nonumber \\ {x}^{\text {other}}_{1} = {x}_{1} + {x}_{1}\odot {g}_{2}, \nonumber \\ {x}^{\text {other}}_{2} = {x}_{2} + {x}_{2}\odot {g}_{1}. \end{aligned}$$

Each embedding vector ${x}_{i}$ exploits discriminative clues obtained from both images in the pair by taking into account its own gate vector and the other image’s gate vector, resulting in two attentive feature vectors, ${x}^{\text {self}}_{i}$ and ${x}^{\text {other}}_{i}$, respectively. Finally, each attentive feature vector, ${x}^{j}_{i}$, is fed to the classification layer that predicts the probability of being in a particular class (i.e., a particular clock score). Mathematically, we have

3 $$\begin{aligned} {p}^{j}_{i} = \text {softmax}(W{x}^{j}_{i}+b),\quad j\in \{\text {self, other}\}, \quad i\in \{1,2\}. \end{aligned}$$

### Model training

**Figure 3:**
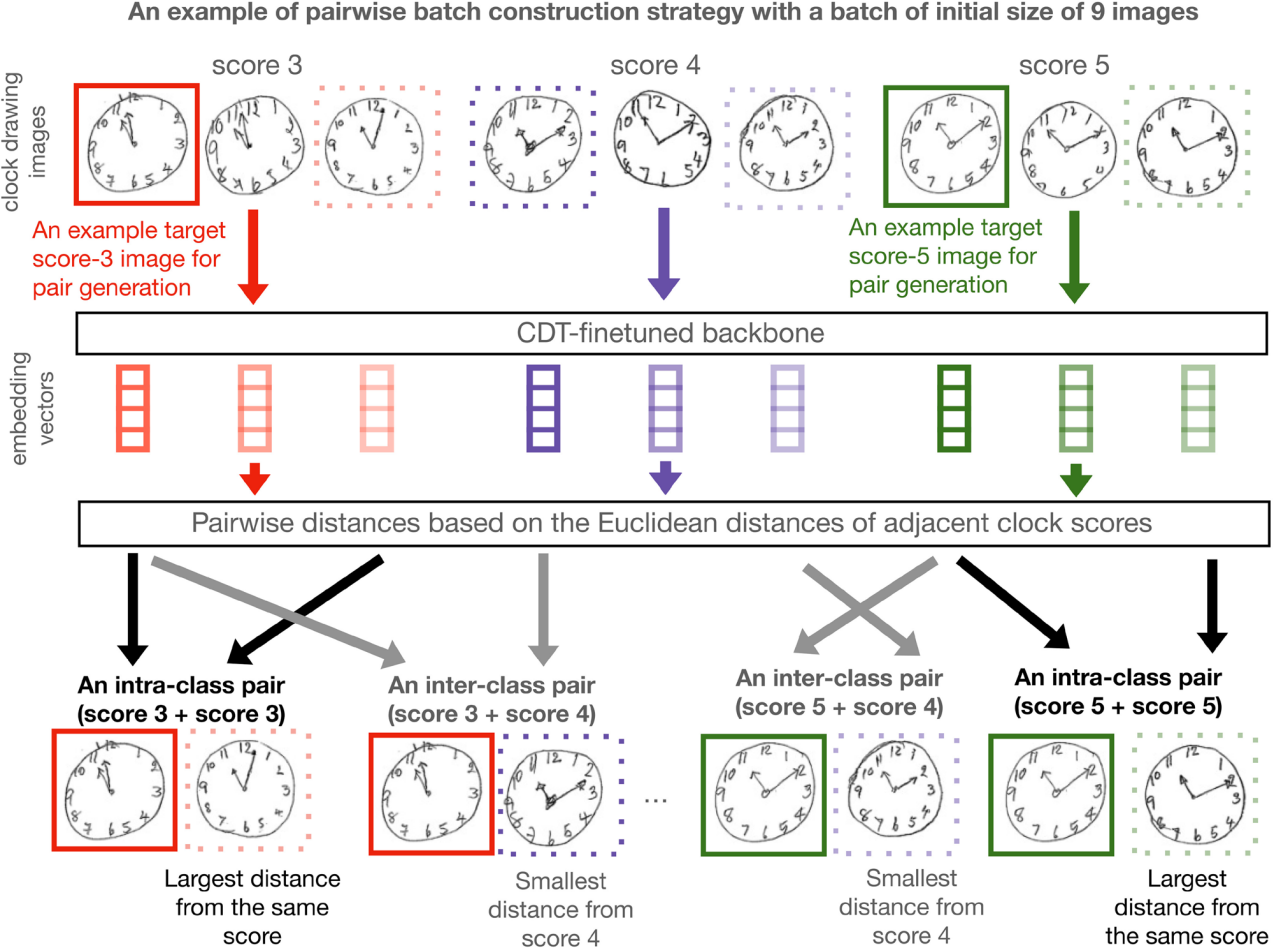
Embedding pairwise batch construction. The backbone model embeds images from all scores and uses them to calculate the Euclidean distance between the image embedding vectors from the same or adjacent scores. The final pairwise batches consist of intra-class and inter-class image pairs that will be used to train API-Net.

To optimize the model’s parameters, the loss function described in Eq. (4) is minimized with respect to the parameters based on the pairwise batch construction procedure demonstrated in Fig. 3.

#### Loss function

The four prediction vectors, ${p}^{\text {self}}_{1}$, ${p}^{\text {other}}_{1}$, ${p}^{\text {self}}_{2}$, and ${p}^{\text {other}}_{2}$, are used to compute the following loss function:

4 $$\begin{aligned} \mathcal {L}&= {\mathcal {L}}_{CE}+\lambda {\mathcal {L}}_{RK}, \end{aligned}$$

5 $$\begin{aligned} {\mathcal {L}}_{CE}&= - \sum \nolimits _{i \in \{1, 2\}}\sum \nolimits _{j \in \{\text {self, other}\}} {y}^{T}_{i}\text {log}({p}^{j}_{i}), \end{aligned}$$

6 $$\begin{aligned} {\mathcal {L}}_{RK}&= \sum \nolimits _{i \in \{1, 2\}}\text {max}(0,{p}^{\text {other}}_{i}({c}_{i})-{p}^{\text {self}}_{i}({c}_{i})+\epsilon ), \end{aligned}$$

where ${{\mathcal {L}}_{CE}}$ is the cross entropy loss, ${{\mathcal {L}}_{RK}}$ is the score ranking loss, $\lambda$ is a regularization parameter balancing the two losses, ${y}_{i}$ is the one-hot encoded ground-truth label vector of image *i*, *T* denotes the transpose of a matrix, ${c}_{i}$ is the correct class for image *i*, and $\epsilon$ is the score-ranking-loss margin.

The cross entropy loss, defined in Eq. (5), is employed to make sure that the model can predict accurate clock-drawing scores using any of the four attentive feature vectors. The score ranking loss is used to encourage the model to prioritize ${x}^{\text {self}}_{i}$ over ${x}^{\text {other}}_{i}$ since ${x}^{\text {self}}_{i}$ is activated by its own gate vector and should intuitively be able to give a more accurate clock score to image *i*, compared to ${x}^{\text {other}}_{i}$, which is activated by the gate vector of image *j*. In particular, the score ranking loss does not penalize the model if the predicted probability of the correct class, ${c}_{i}$, of image *i* obtained from ${x}^{\text {self}}_{i}$ is larger than that obtained from ${x}^{\text {other}}_{i}$ by at least $\epsilon$, as shown in Eq. (6).

#### Embedding pairwise batch construction

We randomly partitioned the whole training set in a stratified manner into *K* batches. For each image ${m}_{i}$ in a batch, we generated two types of image pairs: intra-class and inter-class pairs. Considering only the images within the same batch, to create an intra-class image pair, we combined ${m}_{i}$ with the most dissimilar image within the same class, and, to create an inter-class image pair, we combined ${m}_{i}$ with the most similar image that is in an adjacent class. For instance, for a clock drawing image with a score of four, we generated three image pairs: one intra-class pair that included the most dissimilar image with a score of four, one inter-class pair that included the most similar image with a score of three, and one inter-class pair that included the most similar image with a score of five. The motivation behind this design is that we want to encourage the model to be able to not only recognize a wide variety of images within the same clock score, but also distinguish visually similar images from different scores. We measured the similarity between two images based on the Euclidean distance between the embedding vectors of the two images, which were generated by the backbone. An example of pairwise batch construction is shown in Fig. 3.

### Model inference

To make a prediction on a new image, we pass the image by itself as the input to the backbone to get an embedding vector and then pass the embedding vector directly to the trained classifier to produce the final clock score. This is in contrast to the training phase where we provided a pair of images and the resulting embedding vectors are passed to the API component, followed by the classifier. This procedure is the same as the conventional CNN classification pipeline except that the backbone of API-Net has been specifically trained to be suitable for fine-grained image classification.

### Consent statement

The study was conducted in accordance with the Declaration of Helsinki, The Belmont Report, CIOMS guideline and International Conference on Harmonization in Good Clinical Practice (ICH-GCP) and was approved by the Ethics Committee of Chulalongkorn University (no. 383/2022) on 18th March 2022. For this study, the raw data were first extracted from the hospital information system, All data were collected as a part of routine clinical service, and patients’ identities, including names, patient IDs, and personal information, were de-identified. This approach ensured privacy throughout the research process. No specific consent is needed for statistical analyses of aggregated deidentified data.

## Experiments and results

### Experiment settings

To assess the performance of API-Net on the Shulman clock scoring task, we conducted four main experiments. First, we performed a pairwise batch construction strategy experiment to investigate the effects of different batch construction approaches on the model’s performance. Then, we benchmarked API-Net with the best-performing pairwise-batch-construction strategy to several strong CNN-based baselines including those that have established state-of-the-art performance in the Shulman scoring task^27^. After that, we assessed the performance of the models on a fine-grained binary classification task that only used images with scores of 4 and 5, which were all visually similar. Finally, we performed an interpretability experiment using Score-CAM^36^, one of the most widely-used visualization methods, to generate visual explanations that can be used to support the model’s decision.

All experiments were conducted five times, each with different stratified random training-validation-test split: 50% as training data, 25% as validation data, and 25% as test data. According to Fig. 1, only 3% of all acquired clock images have a score of three or below. To mitigate the class imbalance problem, we relabeled them as having a score of 3. The relabeling process does not prevent our method from being used as a cognitive screening tool for early detection of neurocognitive disorders because such conditions correspond to a Shulman clock score of four (sensitivity 90% and specificity 39%), while Shulman clock scores of less than four correspond to a more severe neurocognitive disorders (with sensitivity 86% and specificity 72–100%)^11^. The data preprocessing and model training pipeline have been implemented in Python and executed on a computer workstation with an NVIDIA TITAN RTX. The parameters of a model were optimized using the adaptive moment estimation (Adam) optimizer^37^. The performance of the models was evaluated using four metrics: classification accuracy, F1-score, precision, and recall.

### Pairwise batch construction strategy experiment

We compared the proposed Embedding pairwise batch construction strategy as described in the “Methods” section to two other strategies: the Raw and Random strategies. The Raw strategy used the same procedure as the Embedding strategy, except that we changed how we measured the similarity between two images. Specifically, the Raw strategy measured the similarity based on the Euclidean distance between the raw pixels of the images, whereas the Embedding strategy measured the similarity based on the Euclidean distance between the embedding vectors of the images that were generated by the backbone. For the Random strategy, we simply paired an image with another image within the same batch at random, regardless of the clock scores. In addition to the three different batch construction strategies, we also experimented with two different sets of backbone’s parameters. The first set was pretrained solely on ImageNet^19^, referred to as the ImageNet-pretrained backbone. The second set was initially pretrained on ImageNet and later finetuned on the clock images from our training dataset for 40 epochs, termed the CDT-finetuned backbone. Given a combination of a batch construction strategy and a trained backbone, we froze the parameters of the backbone and trained the API component and classifier using a learning rate of 0.00005 and a batch size of 60, which contained 20 images from each class, for 100 epochs. $\lambda$ in Eq. (4) and $\epsilon$ in Eq. (6) were set to 1 and 0.005, respectively (see Supplementary Table S.1 for more information on the effect of $\lambda$ and $\epsilon$ on the model’s performance). Finally, to evaluate the influence of the presence of intra-class pairs, we compared the proposed method that used both intra-class and inter-class pairs to that using only the inter-class pairs.

The performance of different batch construction strategies is summarized in Table 1. We conducted an analysis of variance (ANOVA) test at a significance level of 0.05 to compare the mean accuracy of different strategies. With the obtained p-value of 0.0248, we rejected the null hypothesis, which stated that there was no statistically significant difference in the mean accuracy of the different strategies, implying that at least one strategy’s performance is different from the others. After that, we used a paired two-sample t-test to compare the mean accuracy of the proposed strategy, the Embedding strategy with the CDT-finetuned backbone, to each of the 6 remaining strategies listed in Table 1, one at a time, resulting in a total of 6 tests being performed. For each test, the null hypothesis was that the mean accuracy of the proposed strategy was less than or equal to that of the other strategy being compared, and the alternative hypothesis was that the mean accuracy of the proposed strategy was higher than the other’s. The p-values for the Random strategy with the ImageNet-pretrained backbone and CDT-finetuned backbone cases were 0.006 and 0.0113, respectively. The p-values for the Raw strategy with the ImageNet-pretrained backbone and CDT-finetuned backbone cases were 0.0105 and 0.0064, respectively. These p-values have enabled us to reject the null hypothesis, implying that the Embedding strategy with the CDT-finetuned backbone significantly yielded higher mean accuracy compared to the Random and Raw strategies.

**Table 1:** The means and standard deviations of the Shulman score classification accuracies, F1-scores, precisions, and recalls obtained from API-Net with different pairwise batch construction strategies and backbones over 5 different stratified random training-validation-test data splittings.

Batch construction strategy	Accuracy	F1-Score	Precision	Recall
Random (ImageNet-pretrained)	0.7660 ± 0.0069	0.7546 ± 0.0110	0.7640 ± 0.0058	0.7660 ± 0.0069
Random (CDT-finetuned)	0.7606 ± 0.0104	0.7444 ± 0.0135	0.7640 ± 0.0107	0.7606 ± 0.0104
Raw (ImageNet-pretrained)	0.7712 ± 0.0035	0.7645 ± 0.0073	0.7680 ± 0.0027	0.7712 ± 0.0036
Raw (CDT-finetuned)	0.7701 ± 0.0048	0.7613 ± 0.0062	0.7665 ± 0.0044	0.7701 ± 0.0048
Embedding (ImageNet-pretrained)	0.7668 ± 0.0110	0.7564 ± 0.0175	0.7622 ± 0.0057	0.7668 ± 0.0110
Embedding (CDT-finetuned)	0.7802 ± 0.0030	0.7653 ± 0.0084	0.7727 ± 0.0057	0.7743 ± 0.0054
Embedding (CDT-finetuned without the intra-class pairs)	0.7786 ± 0.0121	0.7764 ± 0.0146	0.7780 ± 0.0128	0.7786 ± 0.0121

The p-value for the Embedding strategy with the ImageNet-pretrained backbone case was 0.0345, which was also less than the significance level of 0.05, implying that the proposed Embedding strategy that uses the CDT-finetuned backbone significantly achieved higher mean accuracy than that using the ImageNet-pretrained backbone. The p-value was 0.841 for the Embedding strategy with the CDT-finetuned backbone that were trained without the intra-class pairs case. Consequently, we failed to reject the null hypothesis, implying that there was not enough evidence to support that there was a significant difference between the mean accuracy of the proposed method when the intra-class pairs were included or excluded. Then, we conducted an F-test to assess the variances of the accuracies obtained. The null hypothesis was that the variance obtained from the proposed method with the intra-pairs included was greater than or equal to that trained without using the intra-pairs. According to the obtained p-value of 0.0396, we rejected the null hypothesis, implying that we have enough evidence to conclude that the presence of the intra-class pairs enabled the proposed method to have more consistent performance, as measured by the reduced variance of the accuracy.

In light of these statistical analyses, it is evident that the optimal batch construction strategy is the Embedding pairwise batch construction strategy with the CDT-finetuned backbone trained using both intra-class and inter-class pairs. This method attained a peak mean classification accuracy of 0.78, complemented by an F1-score of 0.77, precision of 0.77, and recall of 0.77. Consequently, we used the Embedding strategy with the CDT-finetuned backbone trained using both the intra-class and inter-class pairs for the remaining experiments.

### Shulman score fine-grained classification experiment

We benchmarked API-Net, which is designed for fine-grained classification, to several ImageNet-pretrained CNN models consisting of VGG16^21^, ResNet-152^22^, and DenseNet-121^23^, which are widely used for general image classification tasks. As these CNN models have established state-of-the-art performance on Shulman score classification^27^, we considered these models as strong baselines for our task. For the baseline models, we used the same data, image preprocessing, and image augmentation, as those used by API-Net for fair comparisons. For API-Net, we experimented with three backbone architectures consisting of VGG16, ResNet-152, and DenseNet-121, reflecting what we have selected as the baseline models. We compared two different settings for the API-Net training process as shown in Supplementary Fig. S.1: backbone freezing and gradual unfreezing. With backbone freezing, the model training is exactly the same as that used in the pairwise batch construction strategy experiment. In other words, we optimized the API-Net’s parameters for 100 epochs while keeping the CDT-finetuned backbone fixed. For the gradual unfreezing case, we updated the API and classifier components without modifying the CDT-finetuned backbone for 10 epochs. After that, we unfroze the backbone and then simultaneously updated all the three API-Net components for 90 epochs.

We assessed the performances of the methods under consideration on the Shulman clock score classification task. Particularly, each clock drawing image had to be classified by the models as having one of the three possible scores: score 3, score 4 and score 5. As shown in Table 2, for the baseline models, DenseNet-121 slightly outperformed VGG16 and ResNet-152, which has also been observed in a prior work^27^. Incorporating the API component to the baseline models along with the gradual unfreezing strategy improved the models’ performances by approximately 2–3%. API-Net with ResNet-152 as the backbone that was trained with the gradual unfreezing strategy outperformed all the methods, achieving a classification accuracy of 0.79, an F1-score of 0.80, a precision of 0.79, and a recall of 0.79.

**Table 2:** The means and standard deviations of the three-class Shulman score classification accuracies, F1-scores, precisions, and recalls over 5 different stratified random training-validation-test data splittings.

Model	Accuracy	F1-score	Precision	Recall
ResNet-152	0.7668 ± 0.0074	0.7581 ± 0.0079	0.7654 ± 0.0080	0.7668 ± 0.0074
VGG16	0.7668 ± 0.0139	0.7608 ± 0.0159	0.7628 ± 0.0151	0.7668 ± 0.0139
DenseNet-121	0.7740 ± 0.0160	0.7708 ± 0.0142	0.7764 ± 0.0183	0.7740 ± 0.0160
API-Net (ResNet-152) with backbone freezing	0.7802 ± 0.0030	0.7653 ± 0.0084	0.7727 ± 0.0057	0.7743 ± 0.0054
API-Net (VGG16) with backbone freezing	0.7799 ± 0.0096	0.7691 ± 0.0130	0.7819 ± 0.0070	0.7799 ± 0.0096
API-Net (DenseNet-121) with backbone freezing	0.7763 ± 0.0070	0.7677 ± 0.0092	0.7750 ± 0.0058	0.7763 ± 0.0070
API-Net (ResNet-152) with gradual unfreezing	0.7892 ± 0.0104	0.7964 ± 0.0120	0.7871 ± 0.0108	0.7892 ± 0.0104
API-Net (VGG16) with gradual unfreezing	0.7807 ± 0.0118	0.7745 ± 0.0104	0.7779 ± 0.0127	0.7807 ± 0.0118
API-Net (DenseNet-121) with gradual unfreezing	0.7828 ± 0.0025	0.7782 ± 0.0016	0.7821 ± 0.0029	0.7828 ± 0.0025

### Shulman score 4 and score 5 fine-grained binary classification experiment

To assess the effectiveness of API-Net in classifying visually similar images, we focused on images with the scores of 4 and 5. For this experiment, ResNet-152^22^ was chosen as both our benchmark and the backbone for API-Net. API-Net was trained with two different settings: backbone freezing and gradual unfreezing. As shown in Table 3, API-Net with backbone freezing achieved a mean classification accuracy, F1-score, precision, and recall of 0.80, outperforming other methods examined. To compare the performance of each API-Net setting to the benchmark, we conducted paired two-sample t-tests. In particular, for each API-Net setting, the null hypothesis was that the mean accuracy of API-Net was less than or equal to that of the benchmark, and the alternative hypothesis was that the mean accuracy of API-Net was higher than the benchmark’s. While we failed to reject the null hypothesis (p-value = 0.7483) for the gradual unfreezing case, we were able to reject the null hypothesis (p-value = 0.0451) for the backbone freezing case, implying that API-Net with backbone freezing significantly yielded higher mean accuracy than the benchmark’s. In this experiment, the gradual unfreezing method’s underperformance might stem from unintentional alterations in the expertly tuned feature extraction layers of the backbone, especially when restricted to a dataset of only scores 4 and 5. The potential lack of comprehensive representation of the Shulman dataset’s intricacies might have played a role. As layers were progressively unlocked, some vital pretrained knowledge could have been compromised, leading to decreased performance. Nevertheless, the choice between gradual unfreezing and backbone freezing for training fundamentally rests on dataset characteristics used for training. Essentially, this is a hyperparameter that requires tuning to best align with the dataset in use.

**Table 3:** The means and standard deviations of the clock drawing images classification between Shulman scores 4 and 5.  Accuracies, F1-scores, precisions, and recalls are calculated over 5 different stratified random training-validation-test data splittings.

Model	Accuracy	F1-score	Precision	Recall
ResNet-152	0.7877 ± 0.0087	0.7855 ± 0.0079	0.7931 ± 0.0029	0.7835 ± 0.0094
API-Net (ResNet-152) with backbone freezing	0.8033 ± 0.0106	0.8013 ± 0.0096	0.8028 ± 0.0011	0.8033 ± 0.0106
API-Net (ResNet-152) with gradual unfreezing	0.7901 ± 0.0117	0.7980 ± 0.0218	0.7899 ± 0.01	0.7901 ± 0.0117

### Interpretability experiment

**Figure 4:**
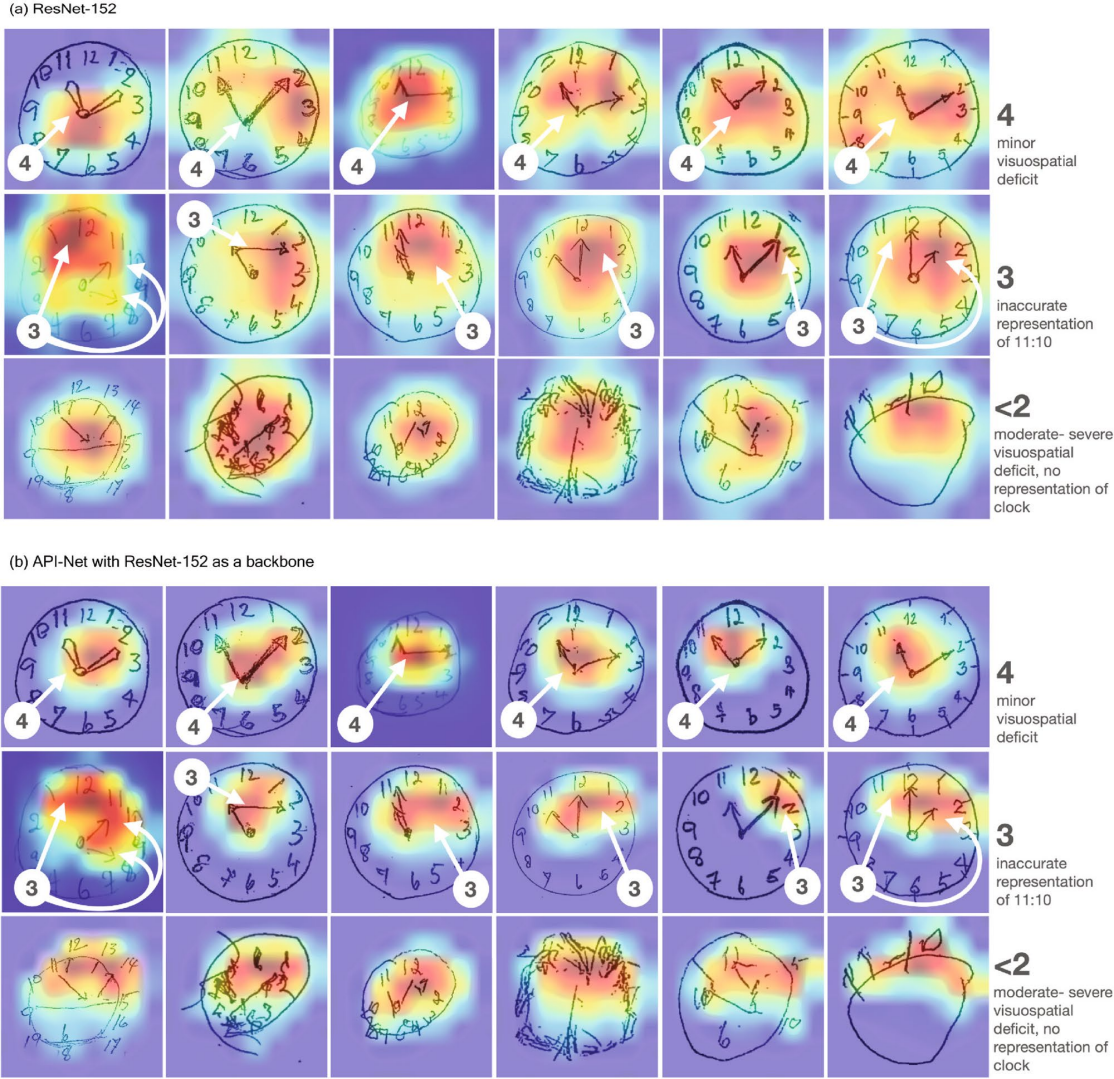
Score-CAM visualization comparisons between (**a**) ResNet-152 and (**b**) API-Net (ResNet-152) with gradual unfreezing. The white arrows in each image pinpoint regions deemed influential to the Shulman score according to physician observations. The Score-CAM visualization from API-Net offers a sharper visual clarity and aligns more closely with the regions indicated by the white arrows. Conversely, the Score-CAM visualization from ResNet-152 seems unfocused, appearing more spread out and farther from the indicated regions. A notable illustration of this is in the 5th column under Shulman score 3 (middle row): The Score-CAM from API-Net distinctly emphasizes the problematic region around the clock’s minute hand, while the Score-CAM from ResNet-152 highlights the entire clock hands. This underscores the superior precision of the API-Net visualization in identifying specific problematic areas.

Presently, clinicians sometimes encounter challenges when scoring clock drawing images that have closely related Shulman scores. This ambiguity also presents in the model decisions, consequently impacting overall performance of the model. To navigate this challenge and make model decisions more transparent, we used a visualization technique called Score-CAM^36^. In this section, we compared Score-CAM visualizations from ResNet-152 and API-Net (ResNet-152) with gradual unfreezing to highlight the superior capability of API-Net in identifying and prioritizing irregularities in clock drawing images, showcasing its enhanced model interpretability. In Fig. 4, Score-CAM visualizations from both ResNet-152 and API-Net are displayed. Though both models accentuate regions exhibiting most of the irregularities in the clock drawing images, the manner in which these areas are highlighted differs significantly between the two. ResNet-152’s Score-CAM, for instance, brings a large expanse around the clock hands into focus for each score, obfuscating a clear understanding of the model’s decision-making process. In contrast, the Score-CAM from API-Net, although highlighting a more confined area, offers a much clearer and intuitive understanding of the model’s rationale. This disparity is particularly pronounced in images bearing a Shulman score of less than 2 (bottom row). In such instances, while API-Net’s Score-CAM adeptly emphasizes areas with notable irregularities and anomalies, the ResNet-152’s version remains fixated on the inner clock circle area. Furthermore, for clock drawing images in the Shulman scores 3 and 4 range (middle and top rows), ResNet-152’s Score-CAM indistinctly highlights both regular and anomalous zones near the clock hands. Contrastingly, API-Net’s Score-CAM specifically zeroes in on the main irregularities, as indicated by the white arrow markers. A striking illustration of this is seen in the image from the 5th column under Shulman score 3 (middle row). Here, the API-Net’s Score-CAM emphasizes a region encompassing the minute hand’s arrow head and a specific number. On the other hand, ResNet-152’s Score-CAM broadly accentuates the clock hands. This nuanced distinction underscores API-Net’s advanced capacity to differentiate between closely similar images, a capability honed through contrastive learning using image pairs.

## Discussion

Deep learning models trained with a standard supervised classification procedure have demonstrated impressive performances on Shulman clock score classification^27^. However, these models sometimes struggle to accurately classify images that are visually alike but have different scores. This challenge becomes especially significant in real-world scenarios where precise differentiation between normal (score 5) and mild (score 4) visuospatial impairment is crucial, given its prevalent occurrence in the general population. Drawing inspiration from human learning patterns, where recognition often arises from comparing two images to discern subtle differences^34^, we applied a contrastive approach to train our model using image pairs.

A crucial aspect of the successful application of API-Net is an effective batch construction strategy. In our experiments, the model using the Embedding pairwise batch construction strategy and CDT-finetuned backbones outperformed all other methods that adopted different strategies (Table 1). As the backbone architecture is purely convolutional, gauging distances in the embedding space, as opposed to the raw pixel space, offers a more resilient metric for image similarity, especially concerning image translation. This underpins the superior performance of the Embedding strategy relative to the Raw strategy (Table 1). Under the Embedding strategy framework, the quality of the backbone used to extract the image embedding vectors greatly affects the model’s performance. Expectedly, the CDT-finetuned backbone, trained using the clock drawing images, yielded better performance compared to the ImageNet-pretrained backbone. While delving into designing an optimal pairwise batch construction strategy with theoretical guarantees is a compelling avenue for future research, it falls outside the purview of this study.

Despite our method’s superiority over the baselines, potential enhancements remain. Since our main focus is on the early detection of neurocognitive disorder such as MCI, we collected the data from a relatively healthy population. As a result, there are fewer individuals who score three or below, resulting in a data scarcity for these lower score categories. Most individuals tend to score within the 4 and 5 range. To address the inherent class imbalance in real-world data, in our study, we consolidated clock drawing images with scores of three or lower under the category of score 3, indicative of severe neurocognitive disorders. It is imperative to amass more data for the under-represented score classes (0–3) to broaden our method’s application across all six Shulman score classes, especially for monitoring purposes.

It is not easy to directly compare our quantitative results to those reported in other studies because of several factors. First, the research goals are different. For instance, while a previous study^27^ has proposed to use Shulman scores for dementia screening, our work used the scores as part of a cognitive screening tool for early detection of neurocognitive disorders, resulting in different score-cutoffs being used for the screening purpose. In particular, a drawn clock must receive a score of 5 (perfect score) to pass our early visuo-spatial deficits screening, whereas a score of 4 would be sufficient to pass the dementia screening test proposed in the study^27^. Since it is much harder to distinguish between images that are scored as 4 and 5 than between images that are scored as 3 and 4, it would be inappropriate to directly use a quantitative metric such as the screening accuracy to compare the methods. Moreover, the studies involved different groups of people with potentially different demographic information and methods of recruitment, making it challenging to compare the results across studies fairly. To facilitate better benchmarking and allow other researchers to directly compare their methods to ours, we have made our codes and dataset publicly available at https://github.com/cccnlab/CDT-API-Network.

Our study underscores the significance of modeling strategy that resonates with the clinical inquiry. To build an effective screening tool for early detection, it is essential to use a modeling strategy that focuses on the nuanced differences distinguishing normal from clinical populations. From the vantage point of API-Net, harnessing contrastive learning to distinguish visually similar images appears to be a promising avenue, especially when dealing with cases that exhibit marginal variations. This perspective can be broadened to encompass other modalities such as speech sounds, fluid biomarkers, and brain imaging. This raises intriguing possibilities for future work in optimizing detection across various medical modalities.

### Supplementary Information

Supplementary Information.

## Data Availability

To foster such a direction and enable a direct benchmarking for interested researchers, we have made our dataset and implementation publicly available at https://github.com/cccnlab/CDT-API-Network.
