# Supplementary material for: Attentive pairwise interaction network for AI-assisted clock drawing test assessment of early visuospatial deficits
Source: Sci Rep. 2023 Oct 23;13:18113. doi: 10.1038/s41598-023-44723-1 (PMC10593802; doi:10.1038/s41598-023-44723-1)
Supplement: Supplementary file 1 — Supplementary Information. [file 41598_2023_44723_MOESM1_ESM.pdf]

Attentive Pairwise Interaction Network for AI-assisted Clock Drawing Test Assessment of Early Visuospatial Deficits

Raksit Raksasat, Surat Teerapittayanon, Kearkiat Praditpornsilpa, Aisawan Petchlorlian, Thiparat Chotibut, Chaipat Chunharas, Itthi Chatnuntawech

Supplementary Information

Investigation of Hyperparameters in API-Net Training

We investigated the impact of the hyperparameters λ in Equation 4 and ε in Equation 6 on the model's performance. For this experiment, we selected the CDT-finetuned ResNet-152 as the backbone. The API-Net models were trained using the gradual unfreezing training approach. We tested λ values of 0.1, 1, and 10 and ε values of 0.0005, 0.005, and 0.05. The means and standard deviations of the classification accuracy are summarized in Supplementary Table S.1.

For each ε value, we performed an analysis of variance (ANOVA) test at a significance level of 0.05 to compare the mean accuracy of the models trained with different λ values. The p-values were 0.6485 for $\varepsilon = 0.0005$, 0.9465 for $\varepsilon = 0.005$, and 0.5768 for $\varepsilon = 0.05$. Consequently, we failed to reject the null hypothesis associated with each case, which stated that there was no evidence of statistically significant difference in the mean accuracy of the models trained with different λ values.

With a fixed λ value, changing ε substantially changed the model's performance. Taking $\lambda = 1$ as an example, when ε was increased from 0.005 to 0.05, the mean accuracy dropped from 0.7892 to 0.7578. We performed a paired two-sample t-test with the null hypothesis which stated that the mean accuracy of the $\varepsilon = 0.005$ case was less than or equal to that of the $\varepsilon = 0.05$ case. With the obtained p-value of 0.0429, we rejected the null hypothesis, implying that using $\varepsilon = 0.005$ significantly yielded higher mean accuracy than using $\varepsilon = 0.05$. Similarly, when ε was decreased from 0.005 to 0.0005, the mean accuracy dropped from 0.7892 to 0.7568. Using a paired two-sample t-test, we also rejected the null hypothesis in this case (p-value = 0.044), implying that using $\varepsilon = 0.005$ significantly yielded higher mean accuracy than using $\varepsilon = 0.0005$.

According to Equation 6, ε defines a margin between probability p^{self} and p^{other}. In particular, it encourages the model to output p^{self} of the correct class that is larger than p^{other} of the correct class by at least ε. The value of ε should be carefully chosen. If ε is too low, the model will not get direct benefits from the contrastive component of the training process. If ε is too high, it will enforce an unrealistically large margin that could adversely affect the model training, especially when the clock drawing images being considered are visually similar. We have observed that using $\varepsilon = 0.005$ worked well in this case. With the desired margin specified by ε, the λ value then determines how much to emphasize on the scoring-ranking loss, which is responsible for enforcing the margin, relative to the cross entropy loss. While we observed that the performance of our model is not sensitive to the choice of λ in this study, we suggest that both ε and λ should be carefully selected, possibly through hyperparameter tuning, when the proposed method is adopted or extended to other tasks and datasets.

λ \ ε	0.0005	0.005	0.05
0.1	0.7501 ± 0.0383	0.7923 ± 0.0149	0.7637 ± 0.0234
1	0.7568 ± 0.0338	0.7892 ± 0.0104	0.7578 ± 0.0282
10	0.7715 ± 0.0225	0.7902 ± 0.0147	0.7737 ± 0.0033

Table S.1. The means and standard deviations of the clock drawing images classification accuracy with different combinations of λ and ε values, calculated over 5 different stratified random training-validation-test data splittings.

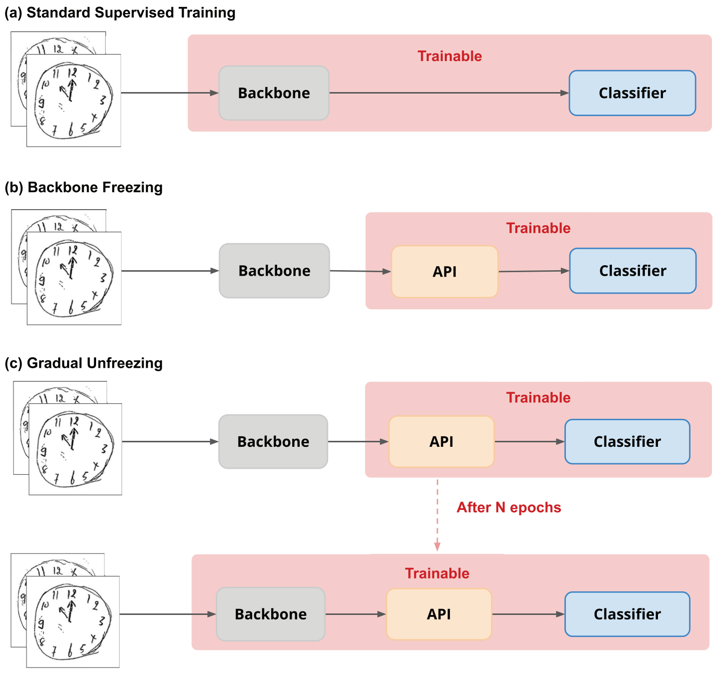

Figure S.1. Different training processes. (a) Standard supervised training for the baseline models. (b) Backbone freezing. The backbone's parameters are not updated during the training process. (c) Gradual unfreezing. Only the API component and classifier are updated during the first N epochs, and then all the components are updated for the remaining epochs.
